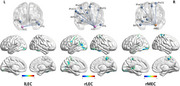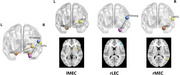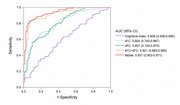# Static and Dynamic Functional Connectivity Alternations of Medial and Lateral Entorhinal Cortex with Subjective Cognitive Decline

**DOI:** 10.1002/alz70856_099610

**Published:** 2025-12-24

**Authors:** Danni Ge, Jiaming Lu, Qian Chen, Futao Chen, Yajing Zhu, Xin Zhang, Bing Zhang

**Affiliations:** ^1^ Department of Radiology, Nanjing Drum Tower Hospital, Affiliated Hospital of Medical School, Nanjing University, Nanjing, Jiangsu, China; ^2^ Nanjing Drum Tower Hospital, Affiliated Hospital Clinical College of Nanjing Medical University, Nanjing, Jiangsu, China

## Abstract

**Background:**

To investigate the static functional connectivity (sFC) and dynamic functional connectivity (dFC) of medial entorhinal cortex (MEC) and lateral entorhinal cortex (LEC) in individuals with subjective cognitive decline (SCD) and the associations with cognitive performance, spatial navigation and olfactory memory.

**Method:**

Seventy‐seven control subjects and 106 SCD individuals were enrolled, and neuropsychological evaluations, 2D computerized spatial navigation test, olfactory memory test and resting‐state functional magnetic resonance imaging (rs‐fMRI) were collected. Bilateral MEC and LEC were selected as seeds to investigate alternations of the volumes, sFC and dFC.

**Result:**

Compared to control subjects, SCD individuals exhibited decreased sFC between bilateral LEC and visual network, between right LEC and left posterior cingulate gyrus and sensory motor network, and between right MEC and left hippocampus, visual network and sensory motor network. The dFC between right LEC and right triangular part of inferior frontal gyrus (IFGtriang) decreased, while dFC between left MEC and right putamen, and between right MEC and right middle temporal gyrus increased. In SCD group, there was a positive correlation trend between the volumes of bilateral MEC and spatial navigation ability, and sFC between right LEC and visual network was positively correlated with olfactory function. The dFC between right LEC and right IFGtriang was correlated positively with global cognitive performance. The combination of sFC and dFC as biomarkers to identify SCD showed an area under curve of 92.1%.

**Conclusion:**

There were functional alternations of EC subregions in SCD individuals, and we demonstrated the association between LEC and spatial navigation, and MEC and olfactory memory. The combination of sFC and dFC may be a new neuroimaging biomarker for the early diagnosis of AD.